# Myeloproliferative Neoplasms: Challenging Dogma

**DOI:** 10.3390/jcm13226957

**Published:** 2024-11-19

**Authors:** Jerry L. Spivak

**Affiliations:** Hematology Division, Department of Medicine, Johns Hopkins University School of Medicine, Baltimore, MD 21287, USA; jlspivak@jhmi.edu

**Keywords:** hematopoietic stem cells, blood volume, MPN driver mutations, diagnostic criteria, natural history, phlebotomy, myelofibrosis, ARCH, CHIP, hydroxyurea, acute leukemia

## Abstract

Myeloproliferative neoplasms, polycythemia vera, essential thrombocytosis, and primary myelofibrosis are a unique group of clonal hematopoietic stem cell neoplasms that share somatic, gain-in-function driver mutations in *JAK*2, *CALR*, and *MPL*. As a consequence, these disorders exhibit similar phenotypic features, the most common of which are the ceaseless production of normal erythrocytes, myeloid cells, platelets alone or in combination, extramedullary hematopoiesis, myelofibrosis, and a potential for leukemic transformation. In the case of polycythemia vera and essential thrombocytosis, however, prolonged survival is possible. With an incidence value in the range of 0.5–2.0/100,000, myeloproliferative neoplasms are rare disorders, but they are not new disorders, and after a century of scrutiny, their clinical features and natural histories are well-defined, though their individual management continues to be controversial. With respect to polycythemia vera, there has been a long-standing dispute between those who believe that the suppression of red blood cell production by chemotherapy is superior to phlebotomy to prevent thrombosis, and those who do not. With respect to essential thrombocytosis, there is a similar dispute about the role of platelets in veinous thrombosis, and the role of chemotherapy in preventing thrombosis by suppressing platelet production. Linked to these disputes is another: whether therapy with hydroxyurea promotes acute leukemia in disorders with a substantial possibility of longevity. The 21st century revealed new insights into myeloproliferative neoplasms with the discovery of their three somatic, gain-of-function driver mutations. Almost immediately, this triggered changes in the diagnostic criteria for myeloproliferative neoplasms and their therapy. Most of these changes, however, conflicted with prior well-validated, phenotypically driven diagnostic criteria and the management of these disorders. The aim of this review is to examine these conflicts and demonstrate how genomic discoveries in myeloproliferative neoplasms can be used to effectively complement the known phenotypic features of these disorders for their diagnosis and management.

## 1. Introduction

Myeloproliferative neoplasms (MPNs), polycythemia vera (PV), essential thrombocytosis (ET), and primary myelofibrosis (PMF) are an unusual group of myeloid neoplasms since they share three gain-of-function somatic driver mutations, *JAK*2, *CALR* and *MPL*, and, as a consequence, there is an inappropriate increase in the production of morphologically and functionally normal red cells, leukocytes, and platelets either together or alone, depending on the particular MPN. There are, of course, the conundrums of how a single MPN driver mutation can cause three different diseases, or how all three driver MPN mutations can cause the same disease. With the advent of the genomic era, there is now a third conundrum of how to meld the lessons of a century of clinical observations with newly acquired genomic insights. The first two conundrums are easily solved, since the three different MPN driver mutations activate the same essential tyrosine kinase for hematopoiesis, JAK2, albeit by different mechanisms, while the phenotypic expression of a specific MPN is not dictated entirely by an MPN driver mutation, but by the host genetic variation, of which sex and age are the most important factors [1]. For example, before the age of 50 years, PV and ET are more prevalent in women, while PMF is uncommon in both sexes; after the age of 50 years, the prevalence of PV and PMF is roughly equivalent in men and women, while ET remains more common in women. The transformation to acute leukemia appears to be more common in men, usually occurring after the age of 60 years, while treatment-related AML has no sex proclivity. The third conundrum, unfortunately, is less easily solved, in part because the clinical lessons of the last century have either been forgotten or never adopted by those educated in this century, and that is the focus of this review, primarily using PV, the commonest MPN, as a surrogate for the MPN as a group, since the same lessons apply to all three diseases.

## 2. Hematopoietic Stem Cell Physiology

**Figure 1:**
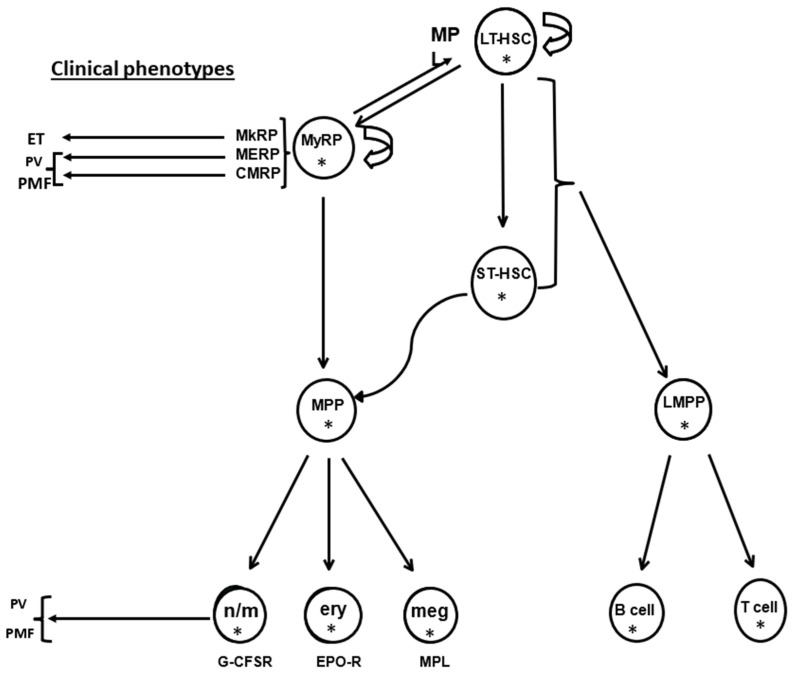
The hematopoietic stem cell (HSC) hierarchy (modified from Yamamoto et al. [14]). HSCs are organized in a hierarchy in which the most primitive HSC, the long-term HSC (CD34+CD38-LT-HSC), is mainly dormant in endosteal bone marrow niches, tethered to osteoblasts through adhesive proteins and thrombopoietin. These LT-HSCs also require the presence of closely opposed megakaryocytes, which maintain HSC quiescence though the secretion of thrombopoietin and CXCL4 [15]. The daily requirement of committed hematopoietic progenitor cells is provided by short-term HSCs (ST-HSCs), which have a limited self-renewal capacity, but a large proliferative capacity. Importantly, the only hematopoietic growth factor receptor expressed by LT-HSCs is the thrombopoietin receptor, MPL. The default commitment pathway for LT-HSCs is to give rise to myeloid repopulating HSCs (MyRPs) with restricted lineage specificity, including megakaryocyte HSCs (MkRPs); megakaryocyte–erythroid HSCs (MERPs), and a common myeloid HSCs (CMRPs), which gives rise to myeloid, erythroid, and megakaryocytic cells. This arrangement reflects the need of LT-HSCs for megakaryocytes as well as thrombopoietin to remain quiescent. It also explains why thrombocytosis is a common presenting manifestation of all three MPNs, and also the pleiomorphic presenting manifestations of MPNs and MPN clonal succession. MPC, multipotent myeloid progenitor cell; LPC, multipotent lymphoid progenitor cell; n/m, neutrophil/monocyte progenitor cell; ery, erythroid progenitor cell; mk, megakaryocytic progenitor cell; B, B-cell progenitor cell; T, T-cell progenitor cell; G-CSFR, granulocyte colony stimulating factor receptor; EPOR, erythropoietin receptor; MPL, thrombopoietin receptor. * Represents the presence of an MPN driver mutation.

MPNs are clonal hematopoietic stem cell (HSC) disorders, since each of the three MPN driver mutations is expressed in a pluripotent, long-term hematopoietic stem cell (LT-HSC). It is important, however, to recognize that, while these three driver mutations give rise to distinct MPN disease phenotypes, there appears to be an ancestral LT-HSC involved, which does not express an MPN driver mutation. This MPN driver mutation-negative LT-HSC was identified when a *JAK*2 V617F mutation-negative leukemic transformation was observed to occur in *JAK*2 V617F mutation-positive patients [2], and that LT-HSC could also be involved when phenotypical PV or ET develops in the absence of an MPN driver mutation (a “triple-negative MPN”). In some triple-negative female ET patients, the *JAK*2 V617F mutation is expressed only in the platelets [3], while others have rare somatic or germline *MPL* mutations [4,5,6,7]. Importantly, in addition to the pluripotent LT-HSC and its classical direct progeny, the short-term HSC (ST-HSC), additional monoclonal or oligopotent HSC (known as myeloid repopulating units or MyRPs) have been identified [8]. These HSCs also are the direct progeny of LT-HSCs, and appear to play a role in MPN pathophysiology, particularly with regard to phenotypic shifts between the three MPNs, making understanding HSC physiology essential for MPN diagnosis and management (Figure 1).

Figure 1 updates our understanding of the classical HSC hierarchy [8], in which the pluripotent LT-HSC presides at the apex and give rise directly to ST-HSCs, which have limited repopulation activity and whose progeny are the committed erythroid, myeloid, and megakaryocytic hematopoietic progenitor cells. We now know that LT-HSC also gives rise directly to three types of myeloid repopulating stem cells (MyRPs): megakaryocyte repopulating cells (MkRPs); megakaryocyte-erythroid repopulating cells (MERPs); and common myeloid repopulating cells (CMRPs), all of which have HSC repopulating activity, like LT-HSCs [8], albeit with some restrictions [9]. Importantly, however, the only hematopoietic growth factor receptor expressed in LT-HSCs is the thrombopoietin receptor (MPL) [10], while the other hematopoietic growth factor receptors, erythropoietin (EPOR), granulocyte (G-CSFR), and granulocyte-macrophage colony stimulating factor receptors (GM-CSFRs), are expressed only in the committed progeny of the various types of HSCs.

The biological significance of MkRPs is due to the important function of their progeny, the megakaryocytes, which keep unneeded LT-HSCs quiescent in their bone marrow osteoblast niches [11]. Indeed, thrombocytosis is the default product of the LT-HSC when stressed [10]. In the absence of marrow megakaryocytes or thrombopoietin, LT-HSCs enter into a cell cycle, leave their marrow osteoblast niches, and either differentiate or migrate from the bone marrow to the circulation. While the physiologic role of MERPs and CMRPs are less clear, they mirror several of the various PV clinical phenotypes. Furthermore, since the G-CSFR usually employs JAK1 [12] as its cognate tyrosine kinase, the CMRP not only ensures a myeloid expression pathway for PV, but also the possibility for phenotypic shifts between the three MPNs [13]. Importantly, as mentioned above, some so-called triple-MPN mutation-negative ET patients, usually women, express *JAK*2 V617F only in their platelets, which, of course, is evidence of the existence of MkRP.

## 3. Phenotypic Mimicry and MPN Diagnosis

### 3.1. Blood Volume Physiology

PV is the only MPN in which the potential risk of a thrombotic event is not only present at diagnosis, but thrombosis may also be the presenting event that leads to the diagnosis of PV [16]. Therefore, diagnostic accuracy is of the utmost importance for distinguishing PV from its companion MPN, and in eliminating the thrombotic risk immediately by phlebotomy. Importantly, erythrocytosis developing in PV differs from erythrocytosis due to hypoxia or secondary erythrocytosis due to an increase in erythropoietin production. Under the conditions of tissue hypoxia or inappropriate erythropoietin production, the increase in the hormone results not only in a surge of erythrocyte production, but also the shrinkage of plasma volume [17]. The latter effect, which is most obvious at high altitudes, is a protective mechanism to prevent blood volume from expanding excessively, and is a cause of fatalities when recombinant erythropoietin is abused by athletes.

In PV, however, where erythropoietin production is suppressed as red cell production increases, there is no plasma volume shrinkage; rather, paradoxically, the plasma volume usually expands, which provides protection from hyperviscosity, but only to a limited degree, while masking the true hematocrit (Hct) [18,19]. Indeed, the clinical scrutiny of PV in the last century is congruent with studies by several future Nobel Prize Laureates to understand blood volume physiology in health and disease [20]. The use of ^51^chromium as a red cell label for red cell mass (RCM) measurement in conjunction with ^131^albumin as a measure of plasma volume (PV) led to the development of a simple method to measure total body Hct (RCM/RCM + PV) by isotope dilution that does not require a direct measurement of peripheral blood Hct using a separate technique [20].

**Figure 2:**
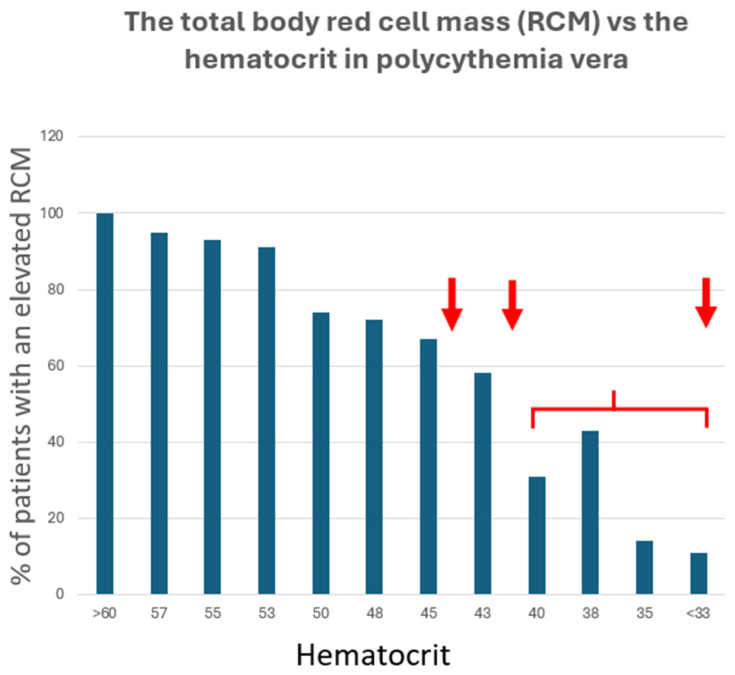
The correlation between the total body red cell mass measured directly and the peripheral blood hematocrit level in PV patients. The data from reference [24] have been replotted to illustrate the important fact that the peripheral blood hematocrit in untreated PV patients fails to correlate with the actual total body hematocrit, even at values falling within the normal hematocrit ranges for normal men and women. As a corollary, even for hematocrits greater than normal, until the hematocrit is greater than 59%, the possibility of a reduced plasma volume still exists (pseudopolycythemia). The red arrows represent the recommended phlebotomy target hematocrits for men and women PV patients, as well as for PV patients with splanchnic vein thrombosis or who are pregnant, respectively.

The reason that an indirect isotopic dilution method was necessary to measure total body Hct is that blood flow through large vessels differs from that in the smaller vessels of the body [21]. Due to the drag of the vessel wall on plasma flow in these small vessels, Hct is actually lower in them because the axial flow of red cells is not impeded. Thus, peripheral blood Hct does not accurately reflect total body Hct, which is lower by approximately 7% [22]. Indeed, because of this, unless peripheral blood Hct is greater than 59%, (Figure 2), the presence of absolute erythrocytosis cannot be guaranteed without performing an RCM/PV study [23].

As an aside, the hemoglobin level (Hgb) is also an imperfect surrogate for an increased red cell mass, despite its recommendation by the WHO [25]. Hgb production is controlled by the red cell and the available iron supply and does not directly relate to blood viscosity. Even Osler and his colleagues understood that Hgb measurements did not accurately reflect the total blood volume [26]. Importantly, since they also did not know about the Hct, they relied on the red cell count, which, while also not correlated with blood viscosity, is still a direct measure of erythropoiesis. Indeed, microcytic erythrocytosis only occurs in thalassemia or in situations causing erythrocytosis [27]. In these two situations, the red cell sacrifices its MCV to maintain its MCHC [28]. Thus, contrary to conventional wisdom, iron is not a rate-limiting feature of erythropoiesis in PV [29].

While the expanded blood volume in PV contributes to hyperviscosity, the blood’s unique physical properties as a non-Newtonian fluid also contribute to increasing blood viscosity. In contrast to Newtonian fluids, where viscosity is linearly related to the shear stress driving flow, blood is non-Newtonian since the relationship of shear stress to viscosity is nonlinear, and also affected by the Hct. When blood flow slows at higher hematocrits due to hyperviscosity, it also becomes even disproportionally more viscous. This is particularly true in the large veins of the splanchnic system, where hepatic vein thrombosis is common in young women with PV, while portal vein thrombosis is more common in men with PV. Indeed, hepatic vein thrombosis is a hallmark of the *JAK*2 V617F mutation [30]. Also contributing to hyperviscosity in PV is nitric oxide scavenging by a high red cell count, which promotes vasoconstriction and platelet adhesion [31].

Importantly, with respect to thrombosis in PV, studies both before and after the discovery of *JAK*2 V617F demonstrated that there is no correlation between peripheral blood Hct and blood volume [32]. Indeed, this is particularly true in women with PV, where the Hct can appear to be normal when hepatic vein thrombosis occurs [18]. This has been characterized as masked erythrocytosis due to the marked increase in plasma volume. Despite this observation, the WHO has eschewed RCM/PV assays for the diagnosis of PV [33]. The importance of this assay historically for the diagnosis of PV is shown in Table 1. The assay also provides a useful measure of how much blood needs to be removed using phlebotomy.

### 3.2. PV Diagnostic Criteria

Since PV is the most common MPN and the ultimate phenotypic expression of the *JAK*2 V617F mutation, diagnostic accuracy is essential to ensure appropriate therapeutic decisions. Importantly, however, as Table 1 indicates, the original WHO PV diagnostic criteria were totally inadequate. Using the surrogates of Hct or Hgb instead of the actual measurement of the red cell mass sacrificed both the sensitivity and specificity in the diagnosis. Using arbitrary values for Hct/Hgb (55.5%/18.5 gm%) for male PV patients and (49.5%/16.5 gm%) for female patients resulted in a stunning rate of diagnostic inaccuracy of 65% for males and 35% for females, according to the WHO algorithm [33,34].

Undaunted, in its second iteration [25] (Table 2), the WHO arbitrarily reduced their Hct and Hgb criteria for both men (>49%/>16.5 gm%) and women (>48%/>16 gm%) PV patients, which now overlaps with healthy male Hct and Hgb values, creating the possibility for men of false-positive results. These new diagnostic criteria reduced the diagnostic inaccuracy to 35% for both sexes but were still unnecessarily high and unacceptable clinically [35]. For comparison, the Polycythemia Vera Study Group’s (PVSG’s) PV diagnostic criteria, which the WHO eschewed, had a diagnostic accuracy of 99% [36]. As such, the current WHO PV diagnostic criteria neglect to capitalize on the accepted pathophysiology of PV with regard to its molecular genetics (see below) and its impact on plasma volume [19], while effectively discouraging the use of an assay essential for the definitive diagnosis of PV.

Inexplicably, the WHO also omitted the only reliable quantitative measurement of red cell production: the red cell count. This denied practitioners the useful diagnostic paradigm of microcytic erythrocytosis [27] and essentially reduced the PV diagnostic footprint to a disease of red cells, when, in fact, it is a clonal HSC panmyelopathy involving red cells, white cells, and platelets. While PV can present with isolated erythrocytosis, this is very uncommon [37]. PV, of course, can also present initially as isolated leukocytosis [24] or thrombocytosis [38], neither of which is included in the WHO PV diagnostic criteria. The WHO also compounded the problem by not warning practitioners that PV can even present with a normal or low Hct due to plasma volume expansion [39], which can be exacerbated by splenomegaly [18]. Of course, remarkably, the WHO also failed to include splenomegaly, which occurs in over 50% of PV patients [33], as a diagnostic criterion.

To further confound the issue of diagnosis, the WHO included a bone marrow biopsy with designated histologic features. Unfortunately, none of these features is specific for PV [40], since they all rely on the presence of activated JAK2, and are also affected by the *JAK*2 V617F mutation quantitative allele burden (MAB, [also known as the variant allele fraction or VAF]) and disease duration. In PV patients with early disease, marrow histology can be normal [41]. Furthermore, there can be marrow fibrosis early in PV, which has no bearing on disease prognosis or marrow function [41]; of course, none of this was acknowledged by the WHO, though it was for these reasons that the PVSG did not recommend the use of a marrow examination for diagnosis in PV [36].

In yet another misstep, the WHO recommended using a highly sensitive *JAK*2 mutation assay, without warning that an exceedingly low *JAK*2 MAB (0.1–1%) can be found in normal individuals [42]. And, of course, approximately 3–5% of PV patients lack a known driver mutation, some of whom may have an *LNK* mutation [43,44]. Finally, the WHO recommended a serum erythropoietin assay as a minor criterion [33]. Unfortunately, this assay lacks diagnostic specificity since it can be normal in PV [32] and low in ET [45].

A recent study using RCM/PV measurements showed a high correlation of RCM elevation with a low serum erythropoietin level, as expected [46], but in the absence of an RCM/PV assay, the serum erythropoietin level is only a reliable surrogate for PV when it is low, and for secondary erythrocytosis when it is high. When the erythropoietin level is normal, neither PV nor secondary erythrocytosis can be excluded [32].

**Figure 3:**
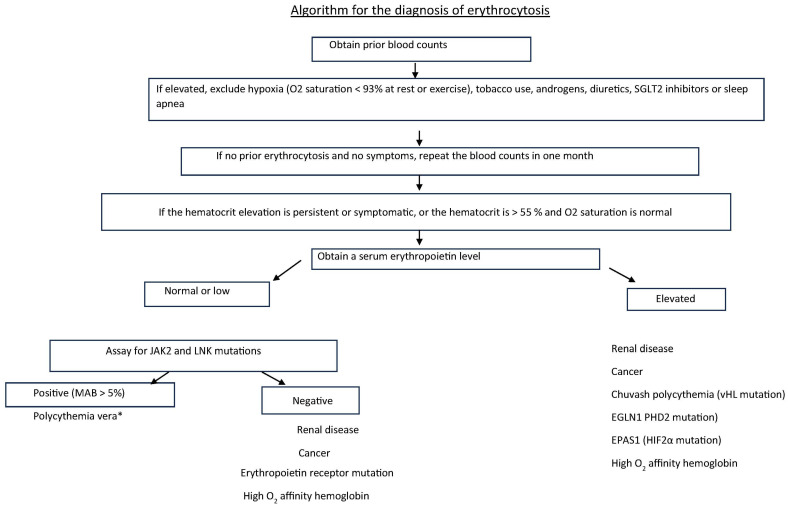
An algorithm for the evaluation of isolated erythrocytosis. In this situation, the frequency of benign causes for an elevated hematocrit or red cell count far outnumbers PV as a cause of isolated erythrocytosis, and an assay for the *JAK*2 or *LNK* mutation is rarely necessary. * this MAB distinguishes PV from CHIP.

When confronted with a high Hct, the physician should first determine how long the abnormality has been present. If such evidence is absent, as shown in Figure 3, and if the patient is asymptomatic, the Hct is less than 55%, [47]; pulse oximetry reveals an arterial oxygen saturation of ≥93%; there is no history of sleep apnea, tobacco, androgen, or diuretic use; and a serum erythropoietin assay is normal, the patient can be observed for a month and the Hct repeated. If the serum erythropoietin level is low, PV should be considered and a quantitative *JAK*2 mutation assay should be obtained; if the serum erythropoietin level is high, secondary erythrocytosis should be considered and a *JAK*2 mutation assay is not necessary [37]. Importantly, if PV is a diagnostic consideration, no Hct level is actually safe from thrombotic risk (Figure 2) [18,39], while in secondary erythrocytosis, apart from hypoxic causes, phlebotomy therapy is not usually necessary.

### 3.3. Natural History of PV

Remarkably, despite over 125 years of observation, the natural history of PV is not well understood with respect to patient longevity and complications, not only because of the unappreciated phenotypic mimicry between ET and PV [48], but also because a consensus on the treatment of PV has yet to be achieved. As Dameshek aptly stated, “*It is difficult to state what the normal course of the disease would be without the various therapeutic methods which undoubtedly influence it*” [49]. In 1950, Videbaek, a Danish hematologist, published the first systematic epidemiologic study of PV involving 125 patients (74 men and 51 women) [50], with an overall survival of 7.3 years for women and 4.5 years for men, while the longest observed patient survival rate was 20 years.

Another epidemiologic study, also from Denmark, published a decade later, involving 250 different PV patients (152 men and 54 women) [51], unfortunately, caught the attention of the then small MPN community, essentially to the exclusion of the Videbaek study cited above, since the later study reported that 50% of untreated PV patients died 18 months after the onset of signs or symptoms, while 50% of patients treated with venesection alone died within 3 years; in comparison, 50% of the patients treated with X-ray were alive 12 years after the first signs of the disease. These authors had evidently not heeded Videbaek’s admonishment, “*Inadequate control and consequently a too casual treatment of the patients are considered to be mainly responsible for the high mortality*” [50].

A more optimistic report within the same time period, and in the absence of X-ray therapy, was reported from the UK, where the median survival was 13.6 years, and not different from PV patients exposed to irradiation [52]. In 1991, an even more positive observation was reported by Rozman et al. from Spain [53]. These investigators evaluated 454 PV and 247 ET patients diagnosed between 1975 and 1987 using PVSG diagnostic criteria, without regard to therapy. They found that, compared to age and sex-specific matched healthy controls, survival was comparable for both PV and ET patients. As a corollary, the survival of PMF patients was markedly reduced.

Surprisingly, as late as 2014, in a US study of 267 PV patients, the median survival was still 13.7 years compared to a cohort of 292 ET patients, whose median survival was 19.8 years [54]. Importantly, however, in an Italian population of 310 PV patients, notably only cited in that report’s supplement, the median survival was 23.8 years, while in 284 ET patients it was 32.7 years, and not statistically different from healthy individuals (*p* = 0.254). The sharp survival differences between the US and Italian studies may be related to differences with respect to diagnosis and therapy, but this, unfortunately, was not discussed in the paper. More recently, another report supported the view that, with appropriate therapy, life expectancy for PV could be normal [55].

Within the context of such chronicity, Rosenthal and Bassen were the first to describe the phenotypes characteristic of the progression of PV [56]. As discussed below, however, we now recognize that such a progression is not inevitable [57], but, rather, is based on host genetic factors, such as sex, age, and acquisition of gene mutations affected by age [58], environment, and therapy [59,60].

### 3.4. Role of Age

Although age has been widely claimed as having an adverse impact on overall survival, particularly ages greater than 60 years old [61], there are other studies [62,63] that refute this contention, since this claim is based on a study [16] involving 1638 patients with a mean age of 65.4 years, of whom 77.5% had cardiovascular disease while 57% were receiving chemotherapy, with no comparison to patients younger than 60 years old. Furthermore, this study’s claims have also been refuted by studies showing that the complications of PV are not different in patients under the age of 40 years when compared to patients older than the age of 60 years [64,65]. Importantly, a study using gene expression also demonstrated that age was not a risk factor in PV patients [57]. Indeed, in the author’s experience, longevity appears to the rule in PV, rather than the exception, even in patients older than 60 years old [66]; in fact, one patient, who developed *JAK*2 exon 12-positive PV at the age of 17 years was alive and well at the age of 71 years, treated with phlebotomy alone.

### 3.5. Role of Sex

In contrast to age, sex, a frequently unmentioned but a very important feature related to MPN prognosis, is a lacking in the most widely published PV risk classification [61] that primarily relies on age and thrombotic phenotype in preference to genotype and sex. Sex is an important feature because women with PV have a genetically different disease in comparison to men [50,57]. They dysregulate fewer genes than men [66] and have fewer cytogenetic abnormalities [67,68], acquire PV or ET earlier than men [69], have a discernably different thrombotic diathesis than men [70], a lower risk of transformation to *JAK*2 V617F mutation-positive myelofibrosis [71], a lower *JAK*2 V617F mutation allele burden than men, and a longer survival than men [72]. PV in women clearly behaves differently than PV in men.

## 4. Role of MPN Gene Mutations

With respect to natural history, epidemiologic data indicate the onset of an MPN increases exponentially with age from birth onward [69]. The biologic basis for this behavior appears to be due to *JAK*2-mutant MPN driver mutations being acquired apparently randomly both in utero and throughout life with an estimated mean latency of 30 years (range, 11–54 years) between driver gene mutation acquisition and diagnosis [73,74]. The penetrance of these somatic mutations is enhanced by age-related clonal hematopoiesis (ARCH) [75], by clonal hematopoiesis of indeterminate potential (CHIP) [76], and by chemotherapy-induced clonal hematopoiesis [77].

Clinically, it was initially surmised that PV evolved monolithically, beginning with an asymptomatic phase followed by an erythrocytosis phase, then a quiescent or spent phase, a postpolycythemic myeloid metaplasia phase, usually with myelofibrosis, and then termination in acute leukemia [56]. This proposal, like the age-associated risk proposal described above, was based on phenotype not genotype, and we now recognize that disease behavior in MPNs is genetically-based, and in part driven by the MPN driver MAB, the important role of which, particularly with regard to *JAK*2 mutations, dictates clinical behavior in PV [78] and PMF [79]. Importantly, myelofibrosis occurring in PV (or ET) does not share the same prognosis that this complication is associated with in PMF [80].

### 4.1. MPN Driver Mutations, MPL, and MPN Disease Behavior

MPNs are clonal stem cell disorders since they start with an HSC acquiring one of the three MPN driver mutations. Importantly, in this regard, MPN driver mutations are not mutually exclusive [81], making the phrase “*BCR-ABL*1 negative myeloproliferative neoplasms” obsolete. MPN driver mutation acquisition can occur any time during life, but the extent to which one MPN driver mutation dominates and when clinical manifestations occur depend on the quantitative driver MAB [82]. This is best understood for the commonest MPN driver mutation: *JAK*2 V617F. Usually, there are no clinical manifestations below an MAB of 5%, which is at the lower end of sensitivity of most clinical MPN driver mutation next-generation sequencing (NGS) assays. Importantly, as discussed below, although MPN driver mutations receive the most attention clinically, disturbed MPL metabolism [83] is an important disease modifier of mutant *JAK*2 in MPNs.

### 4.2. JAK2 Mutations

With respect to the pathogenesis of disease behavior in MPNs, the JAK2 enzyme has two kinase domains, one of which (the JH1 domain) can be activated by binding of the cognate ligands (erythropoietin or thrombopoietin) to their hematopoietic growth factor receptors, while the other, designated the pseudokinase domain (JH2), serves as a negative regulator on the active JH1 kinase domain [84]. As described above, HSCs express only one hematopoietic growth factor receptor: the thrombopoietin receptor (MPL). MPL is a homodimer, with each receptor monomer containing one JAK2 protein. Collectively, when the MPL receptor dimer is not bound to its ligand, thrombopoietin, the JAK2 JH2 pseudokinase domain on each receptor monomer appears to interact with the receptor monomer’s JAK2 JH1 kinase domain, keeping it inactive. When the MPL receptor dimer binds thrombopoietin, the conformation of MPL changes, freeing the two JAK2 JH1 domains from inhibition by their JH2 pseudokinase domains.

When *JAK*2 acquires a somatic mutation, most commonly in exon 14, which encodes its pseudokinase domain, the inhibitory activity of the JAK2 JH1 domain is impaired, allowing the JAK2 kinase domain to phosphorylate MPL and its downstream substrates independent of ligand binding. Infrequently, somatic mutations, deletions, or duplications, can occur in *JAK2* exon 12, the coding region for which is adjacent to the pseudokinase domain, presumably also hindering pseudokinase domain activity, producing unrestricted erythropoiesis and a milder form of PV [85].

PV usually begins as an illness with a low *JAK*2 V617F quantitative MAB that gradually increases at a rate of approximately 1.4%/year [82]. Importantly, with heterozygosity for *JAK*2 V617F, there is still some pseudokinase domain restriction of JAK2 activity, and at the HSC level, heterozygous *JAK*2 V617F-mutant HSCs do not compete well with normal HSCs. Once there are HSCs homozygous for *JAK2* V617F, these HSCs can out-compete normal HSCs. Committed *JAK*2 heterozygous mutant hematopoietic progenitor cells, however, always out-compete both normal HSCs and their progeny, regardless of mutation zygosity [86].

Importantly, in contrast to ET patients, PV patients tend to develop uniparental disomy (UPD) on chromosome 9p, where the *JAK*2 gene is located [87]. UPD is a recombination event resulting in homozygosity for *JAK*2 V617F in formerly *JAK*2 V617F heterozygous mutant cells. Over time, most PV patients develop an increasing *JAK*2 V617F quantitative MAB, only in part due to 9pUPD, which is associated with more active disease. Although there is no direct correlation between the peripheral blood *JAK*2 V617F quantitative MAB and that of the bone marrow HSC, when the peripheral blood *JAK*2 V617F quantitative MAB is >70%, the two are reasonably concordant [88].

The blood quantitative *JAK*2 V617F MAB is the most appropriate assay to monitor targeted therapy against PV HSCs, but the MAB changes very slowly with therapy. In general, PV patients with a *JAK*2 V617F quantitative MAB < 50% have an indolent disease. Recently, a diagnostic algorithm was proposed to define survival in PV and ET [89], but, unfortunately, ET patients were lumped together with PV patients due to the failure to understand that they are genetically different diseases [48], and, of course, the algorithm was inaccurate.

### 4.3. MPL, MPL, and CALR Mutations

Finally, although beyond the scope of this review, it is worth indicating that the thrombopoietin receptor, MPL, can have both germline (*MPL* S505N) and somatic mutations, such as *MPL* 515K/L/A/R, S505N, and S2O4P, causing an MPN [4,6,90], either ET or PMF, as well as benign mutations, such as *MPL* Baltimore (K39N) [91]. Mutations in the thrombopoietin gene [92] also cause benign thrombocytosis, although leukemic transformation and myelofibrosis have been observed with this mutation [93].

*CALR* MPN mutations have an entirely different mechanism of activating JAK-STAT signaling, which involves presenting MPL itself at the cell surface either activated [94] or capable of interacting with thrombopoietin. In this regard, it is important to point out that, in the MPN, MPL is incapable of being glycosylated completely, but although immature, it is still capable of reaching the cell surface [95], where it can be activated by thrombopoietin. The failure of glycosylation also results in the reduced expression of MPL [96,97], which tracks with an increase in the *JAK2* V617F MAB [83], and actually tracks better with the MPN disease phenotype than the *JAK2* V617F MAB [3].

A corollary of impaired MPL processing in the MPN is an elevation of plasma thrombopoietin [96]. Thrombopoietin production is constitutive and normally dependent on metabolism by circulating platelets to maintain its plasma level within the normal range and avoid the pathologic consequences of excess thrombopoietin, such as myelofibrosis [98]. In the MPN, of course, platelet MPL expression is impaired [96] and the degree of impairment differs in the three MPN disease phenotypes according to their *JAK*2 V617F MAB. ET has the mildest phenotype since its *JAK*2 V617F MAB is always less than 50% [72] and its plasma thrombopoietin level is usually within the normal range, whilst in PV and PMF, the plasma thrombopoietin levels are higher than normal [96].

In fact, the prevention of thrombopoietin expression in a transgenic mouse model of PV [99] eliminated the PV phenotype [100], including splenomegaly, myelofibrosis, osteosclerosis, erythrocytosis, leukocytosis, and thrombocytosis, even though the *JAK*2 V617F mutation was still expressed. This was not observed in a transgenic mouse model of ET [101] because *JAK*2 V617F MAB was low, as is typical in human ET [71]. The proposed mechanism for phenotypic correction in the PV murine model is that, in the absence of sufficient megakaryocyte thrombopoietin and CXCL4 production, MPN HSCs cannot remain quiescent in their marrow niches and either differentiate or are trapped in the spleen [15,100]. Thus, PV can be considered essentially as an endocrinopathy, which is important to contemplate when thinking of how best to treat it.

### 4.4. The Management of PV

Like the natural history of PV, the management of PV has been a subject of controversy due to a misunderstanding of the roles of phlebotomy and chemotherapy. Dameshek again described the situation aptly, “*There is a tendency in medical practice—by no means limited to hematologists—to treat almost any condition as vigorously as possible. In hematology, this consists in attempting to change an abnormal number—whether this number is the hematocrit, white cell count, or platelet count to get normal values, whether the patient needs it or not!*” [102].

To begin with the controversy over phlebotomy, Videbaek was the first to recognize that phlebotomy therapy must be assiduous [50], if the benefits of phlebotomy in PV to physically reduce red cell mass to prevent hyperviscosity, to deny iron to red cells to slow their production, and reduce excess blood volume to prevent thrombosis, are to be achieved.

Pearson was the first to define safe target hematocrits for both men and women [103], which have been sadly neglected more recently for women [104], whose blood volume is smaller than men because their androgen production is lower. The possibility of masked erythrocytosis is also most relevant in women [18], and, in the case of hepatic vein thrombosis, the Hct must be reduced by phlebotomy to below 33%, if recurrent thrombosis is to be prevented [66].

Pregnancy also demands Hct reduction to 33% [66], as opposed to the current unvalidated recommendation of <45% [104], which has no physiological basis, while the fetus always receives its iron. As a corollary, hydroxyurea is not an anticoagulant, nor are the leukocyte [105,106] or platelet counts [107] appropriate targets to prevent thrombosis in PV. A fear of inducing iron deficiency with phlebotomy is also misplaced, since iron deficiency in the absence of anemia in adult PV patients does not cause an aerobic impairment [108].

Occasionally, if patients complain of glossitis, dysphagia, or pagophagia [109], a small amount of oral iron will correct these symptoms without causing an abrupt increase in the Hct. An increasing need for phlebotomy means either that the PV erythrocytes have too much iron available to them [110], or that conversion by 9pUPD to *JAK*2 V617F homozygosity has occurred; rarely are PV patients also heterozygous for the HFE gene mutation, *C*282*Y*, which further promotes iron absorption and enhances erythropoiesis.

The routine use of aspirin in asymptomatic PV (or ET) patients without evidence of coronary artery disease or tobacco use has no proven rationale [111], and the use of aspirin in such patients over the age of 60 years is hazardous because of the risk of hemorrhage with no evidence of the benefit of preventing thrombosis [112,113]. Indeed, hydroxyurea was not better than anagrelide for preventing thrombosis due to thrombocytosis [114], and not better than aspirin [115] either.

The recent recognition that a hepcidin mimetic can reduce the phlebotomy requirement in PV [116] is relevant with respect to patient quality of life, and possibly a reduction in thrombotic risk, but while hepcidin reduces iron absorption, it does not reduce iron stores; it simply deprives erythrocytes from recycled iron. So, there is initially a mandatory need for phlebotomy therapy to reduce body iron stores that reside in the red cell mass, not the bone marrow.

Additionally, the largely unrecognized effects of iron depletion by phlebotomy, which a hepcidin mimetic cannot accomplish, include impairing hematopoietic cell cycle activity by inhibiting p21^CIP1/WAF1^ and cyclin D1 expression, impairing DNA synthesis by inhibiting ribonucleotide reductase, which has an iron–tyrosyl free radical center essential for its activity, and promoting apoptosis also by inhibiting p21^CIP1/WAF1^ as well as upregulating *NRG-*1 and *TP*53 expression [117].

While phlebotomy is the most effective way to immediately reduce thrombosis risk, it does not always provide relief from pruritus or erythromelalgia; aspirin is usually effective for the latter, but pruritus may be intractable to standard remedies and myelosuppression may be necessary. This is also necessary to prevent or reduce splenic enlargement. Before the development of targeted therapy for the suppression of activated JAK2 [118] or PV HSCs [119], chemotherapy, ^32^P, or irradiation were the only available treatments.

Within 10 years after the introduction of ^32^P, the suspicion of its leukemogenicity was raised [120]. The debate initially was directed at whether ^32^P also improved survival [121]. This was resolved by a landmark study of PV patients at Johns Hopkins, albeit retrospectively, which showed that neither chemotherapy, X-ray, nor ^32^P prolonged the PV patients’ survival [122], while no leukemia was found in unexposed PV patients, nor was their survival reduced.

Although these observations should have been sufficient to abrogate the routine use of these three therapies in PV, the proponents of marrow suppressive therapy remained adamantly opposed to phlebotomy therapy. In the words of one such proponent, Louis Wasserman, “*Patients with erythremia treated by repeated venesection so as to induce iron deficiency become severely incapacitated and ‘polycythemic cripples’ of little use to themselves or their families*” [108].

Accordingly, the Polycythemia Vera Study Group (PVSG), was formed to conduct a randomized, controlled, clinical trial (PVSG-01) to definitively settle this issue. Unsurprisingly, some members of the PVSG voted against a phlebotomy control arm, but wiser heads prevailed [123]. The actual clinical trial study was, unfortunately, compromised because the initial phlebotomy target was 50% [124], though this was changed to 45% part way through the trial [107].

The PVSG-01 trial was stopped before its completion because of an increasing incidence of acute myelogenous leukemia in the chemotherapy arm [107]. Importantly, these results were similar to the retrospective Hopkins’ study described above with respect to the unacceptable occurrence rate of acute leukemia in the treatment arms as compared to the phlebotomy-only arm. In the PVSG-01 trial, there was, however, an increase in the incidence of thrombosis in the phlebotomy-only arm during the first three years of the trial, presumably coinciding with the time period when the target hematocrit was 50%. Thereafter, there was no difference in the thrombosis rate amongst the three arms and, at the conclusion of follow up, survival was superior in the phlebotomy-only arm. No correlation between the platelet count and thrombosis was identified either. Importantly, in a subsequent retrospective study of 1213 PV patients comparing those who received chemotherapy with those who did not, cancer-free survival was superior in the patients not exposed to chemotherapy [125].

These results did not deter the PVSG chemotherapy proponents from using chemotherapy. This time they chose to examine hydroxyurea (HU), a rapid acting oral inhibitor of DNA synthesis, which inactivates ribonucleotide reductase, the enzyme converting ribonucleotides to deoxyribonucleotides [126]. In PVSG-08, a nonrandomized, open-label, observational study, they compared HU in both therapy-naïve and partially-treated PV patients to the previous phlebotomy-only, flawed control arm of PVSG-01, thus preventing any meaningful comparisons. The results published in a nonpeer-reviewed journal [127] revealed three cases of acute leukemia in 51 therapy-naive PV patients, which was thought to be comparable to the two cases of acute leukemia in the 134 patients in the PVSG-01 phlebotomy arm, and HU was declared safe and efficacious in PV, even though PVSG-08 had violated established clinical trial practice. Remarkably, HU became the accepted method of myelosuppression in PV even after the introduction of therapy specifically targeting *JAK*2 mutations in dividing committed progenitor cells and PV HSCs.

## 5. Myelofibrosis

Myelofibrosis (“MF”), a histologic reaction, is the most misunderstood complication associated with MPNs. It is a reactive, reversible tissue reaction due to diverse stimuli, including inflammation, infection, neoplasia, and tissue damage from a variety of causes, both benign and malignant [128]. Part of the confusion was caused by the erroneous concept that myelofibrosis per se is a distinct disease by extrapolation from the clinical course of primary myelofibrosis (PMF), when it is actually only a reaction of normal (nonclonal) marrow stromal cells to that disease and not vice versa [129,130]. This conflation was further compounded by the promiscuous use of the acronym “MF” to define both the histologic reaction and PMF, which has led to the conceit that targeting the histologic reaction should be an effective method for treating PMF or the other MPNs complicated by it.

Unfortunately, nothing could be further from the truth; since an MPN is a clonal HSC disorder, it is a defective HSC, which is responsible for stimulating normal (i.e., nonclonal) fibroblasts to produce excess collagen [57]. There was no such confusion in the last century, when it was clearly demonstrated that the most severe grade of marrow fibrosis did not disturb hematopoiesis in PV [131], and yet, even in this century, similar data have also been published without dispelling the myth of “MF” [132,133,134]. Myelofibrosis is also part of the natural history of PV (and ET), and although it is usually seen late in these diseases, it can be present early in PV without affecting prognosis [41]. Importantly, it has also been established that myelofibrosis affecting *JAK*2 mutation-positive PV or ET does not have the same prognosis as classical *JAK*2 mutation-positive PMF [80]. Unfortunately, in some “MF” clinical drug trials, this important difference is ignored, which could provide an erroneous estimate of drug efficacy.

Moreover, such is the influence of conventional wisdom and belief in phenotype in MPNs that the WHO has recognized a new syndrome: “prefibrotic primary myelofibrosis”, or pre-PMF. Suffice to say this clinical entity is totally misleading since, first, it could not be confirmed independently [40,134], and second, the analysis of the proposed cases of pre-PMF show that many of these patients actually had PV [135,136,137]. Sadly, the emphasis on a reactive, reversible reaction ignores what is truly prognostically important for MPNs, the *JAK*2 mutation MAB [138,139], and the associated presence of potentially deleterious nondriver mutations and cytogenetic abnormalities [140,141,142] with which they are associated. In this regard, *CALR*-mutation-associated PMF appears to have a longer survival than *JAK*2 V617F-associated PMF [139,143].

Finally, since there is no diagnostic MPN bone marrow histology [40], pathologists have essentially focused on myelofibrosis per se and grading its extent to virtually the exclusion of assessing other important diagnostic clinical findings, even though these are independent of the degree of fibrosis [144]. This was emphasized in two recent clinical trials, one using the JAK1/JAK2 and ACVR1 inhibitor, momelotinib, in PMF, and PV or ET MF [145], and the other employing ruxolitinib in PMF patients [146], where it was found that that there was no correlation between various markers of clinical improvement and changes in marrow fibrosis or its extent.

### Transformation to Acute Myelogenous Leukemia

An unresolved issue in MPNs is whether the evolution to acute leukemia is part of the natural history of these diseases, although this issue pertains more to chronic-phase PV and ET than it does to PMF, with its leukemic transformation rate of 20–30% [147]. Obtaining objective estimates of the incidence of AML in PV, however, is vitiated by the conflation of PV with PMF diagnostically [139]. More importantly, as discussed above, the inappropriate widespread use of radiotherapy or chemotherapy in the MPN has also prevented answering this question, since these therapies by definition are themselves leukemogenic [59].

Unfortunately, this problem has been compounded by the refusal of many clinicians to acknowledge that hydroxyurea, the most widely used chemotherapeutic drug for MPN therapy, is not only leukemogenic, but also ineffective in improving survival or preventing venous thrombosis when compared to either aspirin [115,148] or anagrelide [114,149].

Importantly, part of the problem is the failure of physicians to understand the correct use of phlebotomy therapy [104], or for harboring an unwarranted concern for leukocytosis and thrombocytosis as risks for thrombosis, when they are not [104,105,106]. These situations have led to the uncritical acceptance of hydroxyurea as safe, despite the scientific evidence showing the contrary.

In one retrospective publication, widely cited as proof of hydroxyurea safety, the duration of observation was insufficient as was the number of hydroxyurea-exposed patients to come to any meaningful conclusion [150]; stated differently, “the absence of evidence is not evidence of its absence” [151]. In three more recent publications [147,152,153], evidence inculpating hydroxyurea as leukemogenic was cited [154], but was either ignored or misinterpreted.

Another part of the problem is the reliance on the observation that hydroxyurea is safe because it is widely used for treating sickle cell anemia (SCA), where acute leukemia is rare. SCA, however, is not a clonal HSC disorder. Moreover, the argument that hydroxyurea is a safe drug is rebutted by the fact that it selects cells with a 17p deletion, which renders *TP*53 haplodeficient [155], while at the same time impairing TP53 activation [156], thus allowing *JAK*2-mutant HSCs with other deleterious mutations or DNA damage to pass through the cell cycle and out-compete normal HSCs.

Importantly, in contrast to most treatment-related leukemias [157], the onset of acute leukemia secondary to hydroxyurea in MPN patients is usually delayed for over a decade [158]. Therefore, the physicians who prescribe the drug are not likely to see its consequences, a scenario similar to that of radiation or chemotherapy [159].

This was brought into sharp focus recently with a report of treatment-related leukemia developing in three adult SCA patients who developed acute myelogenous leukemia following a failed bone marrow transplant [160]. All three had previous exposure to hydroxyurea as children, and two were found to harbor a *TP*53 mutant HSC clone before transplantation, which expanded afterward in the settings of immunosuppression and inflammation, while the third, on whom next-generation sequencing (NGS) studies were not performed, had a 7q deletion, which is associated with treatment-related acute leukemia [59].

Acute myelogenous leukemia was, of course, well documented in MPN patients who were never exposed to either radiotherapy or chemotherapy, but the exact frequency of this is unknown because the duration of most observational studies was not long enough [107,161]. Acute leukemia in the absence of drug exposure could be due in part to age-related clonal hematopoiesis (ARCH) [162] or clonal hematopoiesis of indeterminant potential (CHIP) [76], which is not age-restricted, and, in part, due to marrow fibrosis, an inflammatory process, which occurs in approximately 15% of PV patients [80].

A peculiar feature of leukemic transformation in some *JAK*2 V617F-positive MPN patients is its occurrence in a *JAK*2 V617F-negative HSCs [2,163]. In contrast to PMF or PV with myelofibrosis, these *JAK*2 V617F-negative HSC leukemias usually arise during the chronic phase of PV or ET, are often associated with a *TP*53 mutation and hydroxyurea therapy, and are extremely difficult to treat. *TP*53 is, of course, one of several gene mutations, including *TET2*, *DMNT*3a, *ASXL*1, and *JAK*2 V617F that are commonly acquired in an age-dependent fashion [164], and some are also frequently treatment-related [59].

A largely unappreciated feature of PV is that chemotherapy can either damage PV HSC DNA or promote the clonal growth of HSCs that ordinarily cannot out-compete undamaged HSCs [59], while presenting the phenotypic appearance of effectiveness by improving blood counts, which does not influence survival [165]. The latter is seen most dramatically when PV emerges after treatment for acute leukemia [166], chronic myelogenous leukemia [167], or autologous marrow transplantation [160,168].

Equally disturbing is the observation that hydroxyurea resistance or intolerance was associated with an expansion of existing clonal mutations and the emergence of new deleterious mutations in *TP*53, spliceosomes, and chromatin-modifying genes [169], and this was also true for hydroxyurea induced cytopenia [170]. Importantly, hydroxyurea is also capable of altering the DNA copy number [171] and inducing the production of reactive oxygen species, a creator of an inflammatory milieu [172], while impairing DNA synthesis.

Importantly, AML complicating PV comes in the same AML varieties as are observed in the general population: primary or de novo, secondary, and treatment-related [173,174]. These three entities can be broadly distinguished by their molecular and cytogenetic markers. Since the peak incidence of MPNs occurs after the age of 60 years, as does the incidence of de novo AML [69], transformations in some MPN patients are clearly age-related, implicating a role for ARCH, since its incidence also peaks at this time [175]. The presence of some mutations in splicing genes, such *SRSF*2 or *USAF*1, which define secondary leukemia, usually occur in PMF, suggesting that PMF may be thought of as the myelodysplasia of MPNs.

The most important issue with regard to the possibility that leukemia is a feature of the natural history of PV and ET is that not only is mutated *JAK*2 one of the CHIP or ARCH mutations, but it is also the cognate tyrosine kinase of another oncogene, *MPL*, and facilitates its oncogenic behavior as well as that of CHIP mutations, such as *TP*53. Additionally, *JAK*2 V617F has been thought to promote genomic instability by stimulating a homologous recombination [176] and through reactive oxygen species production causing double-strand DNA breaks and abnormal DNA repair [177].

Against these data, however, are the observations that ET and PV patients can have a normal life span, and that cross-sectional studies employing high-resolution SNP arrays [155,178] have demonstrated that, when 9pUPD was excluded, JAK2 V617F-positive and -negative chronic phase MPN patients did not differ greatly with respect to chromosomal aberrations, and usually had fewer than three mutations, with many patients only expressing only *JAK*2 V617F [81,178]_._

MPN disease progression and transformation to acute leukemia were associated with the acquisition of new mutations and chromosomal aberrations, not only UPD, but with changes compatible with contiguous gene syndrome regions other than those on chromosomes 5q and 7q [155,178]. These changes did not appear to be the consequence of *JAK*2 mutational status or its MAB, but rather appeared to be due to patient age, since disease duration was also not associated with them [178], a conclusion supported by other data [141,179,180].

## 6. Going Forward

As we enter the second quarter of the 21st century, our ability to treat MPNs has been substantially increased by the addition of three new JAK2 inhibitors, fedratinib, pacritinib, and momelotinib, each with pharmacologic characteristics differing from ruxolitinib. The FDA has also recently approved a long-acting pegylated interferon that selectively targets MPN HSCs. Additional promising candidate drugs are also in the clinical trial pipeline. Obviously, a major challenge now is how to integrate these various drugs to alleviate patient symptoms and improve overall survival. The latter challenge is most immediate in PMF for which stem cell transplantation is the only potentially curative option.

For PV and ET, the challenges are different but equally important. We are currently unable to consistently distinguish clinically between PV and ET because of the inaccuracies of the WHO diagnostic criteria, and also to treat them safely and effectively because of historically inaccurate biases regarding the appropriate use of phlebotomy and chemotherapy. Furthermore, PV and ET are genetically different illnesses [48,181,182], and it is time to recognize this for our patients’ benefit. It is also imperative that we abandon the use of phenotype to identify which PV and ET patients are at risk of progression [61]. We have the genomic tools to perform this. Age alone is not a risk factor [57], since both young and old MPN patients suffer the same complications.

It is also important to use treatment-naive chronic-phase MPN patients for drug trials. PVSG-01 is the only randomized, controlled drug trial that used well-documented treatment-naive PV patients, save only for phlebotomy therapy, making it a direct, conflict-free referendum on the safety and effectiveness of chemotherapy or ^32^P, both of which failed [107], while also corroborating the retrospective Johns Hopkins study [122]. This approach is parsimonious, considering that MPNs are orphan diseases for which we need unequivocal results.

Problems also exist with respect to MPN therapy. MPN HSCs are resistant to killing by conventional chemotherapy, including hydroxyurea, but they are not resistant to drug-induced DNA damage or the expansion of deleterious HSC clones in the presence of *JAK*2 V617F [183]. Hydroxyurea is being used essentially as a crutch, which is indefensible in two diseases, PV and ET, which are compatible with a normal life span if managed properly. Stated differently, we now have a nonmyelotoxic, targeted therapy, pegylated interferon alpha, which can achieve myelofibrosis-free and overall survival outcomes [184].

Also, with respect to PV, the role of phlebotomy is still totally misunderstood. Yet, it is the only proven way to prevent thrombosis both immediately and in the long term. The introduction of hepcidin mimetics or the enhancement of hepcidin expression in PV patients could provide a welcome respite from phlebotomy, but only after an adequate reduction in body iron stores by phlebotomy.

No chemotherapy drug, including hydroxyurea [185], is antithrombotic; these drugs are only cosmetic and myelotoxic. It is imperative that the FDA removes its irrational mandate that ruxolitinib can only be used in PV, when there is an inadequate response or intolerance to hydroxyurea, particularly when it has been demonstrated these are the very patients who develop deleterious mutations with hydroxyurea exposure [169]. The therapeutic use of the newer JAK2 inhibitors also needs to be broadened by the FDA beyond PMF to meet the needs of chronic-phase PV and ET patients who are either intolerant or refractory to ruxolitinib, which itself can promote *RAS* mutations [186].

At this point in time, pegylated interferon is the most important MPN therapy because of its selective MPN HSC targeting and survival effects, but when and how best to use it alone or in combination with a nonspecific JAK2 inhibitor are still unclear for these chronic diseases with long survival. Taking a lesson from *JAK2* V617F-positive ET, if we can keep *JAK2* V617F MAB less than 50% in PV, we may not need do anything further, so long as the appropriate sex-specific hematocrit values are maintained, since prolonged interferon therapy has its own deleterious organ-specific effects.

Finally, with respect to the issue of acute leukemia in MPNs, it is apparent that, currently, we have no therapy for CHIP or ARCH, but we do have a clear choice about avoiding the accumulation of deleterious genomic changes associated with every chemotherapeutic drug, including hydroxyurea, and also the choice not to hide behind statistics [161] but to avoid creating them.

## Figures and Tables

**Table 1:** Phenotypic mimicry associated with polycythemia vera: the role of both red cell mass and plasma volume measurements in ensuring diagnostic accuracy.

**Patient 1**
A 48-year-old woman with a diagnosis of primary myelofibrosis was referred for a preoperative evaluation for a splenectomy. The hematocrit was 39%, the leukocyte count was 18,000/mL, and the platelet count was 840,000/mL. Because of a normal hematocrit in the presence of a massive splenomegaly, red cell mass and plasma volume studies were performed. The red cell mass was 52 mL/kg with an expected range of 20–30 mL/kg, while the plasma volume was 71 mL/kg with an expected value of 40 mL/kg, indicating the presence of polycythemia vera with the erythrocytosis masked by plasma volume expansion. The patient was phlebotomized to a hematocrit of 40% before surgery.
**Patient 2**
A 49-year-old man was referred for the evaluation of thrombocytosis without splenomegaly. The hematocrit was 45%, the leukocyte count was 10,100/mL, and the platelet count was 781,000/mL. Red cell mass and plasma volume studies were performed since essential thrombocytosis is more common in women. The red cell mass was elevated at 40.4 mL/kg with an expected range of 25–35 mL/kg, while the plasma volume was 41.8 mL/kg, with an expected value of 40 mL/kg, indicating that the patient had polycythemia vera and not essential thrombocytosis.
**Patient 3**
An asymptomatic 61-year-old woman was referred for the evaluation of thrombocytosis. The hematocrit was 44%, the leukocyte count was 12,700/mL, and the platelet count was 799,000/mL. *BCR-ABL* FISH was negative; a *JAK*2 V617F assay revealed a mutation allele burden of 35% and the serum ferritin was 33 mg/mL. Red cell mass and plasma volume studies revealed a red cell mass of 38.5 mL/kg, with an expected range of 20–30 mL/kg, while the plasma volume was 47.1 mL/kg, with an expected value of 40 mL/kg, indicating the patient had polycythemia vera as opposed to essential thrombocytosis.
**Patient 4**
A 68-year-old woman was evaluated for an increase in abdominal girth. A CT scan revealed an enlarged caudate lobe and nodularity of the liver with ascites consistent with hepatic vein thrombosis. The hematocrit was 48.7%, the hemoglobin was 15.2 gm%, and the platelet count was 397,000/mL. The red cell mass was elevated at 43 mL/kg, with an expected range of 20–30 mL/kg. The plasma volume was 43 mL/kg with an expected value of 40 mL/kg, indicating that polycythemia vera was the cause of the hepatic vein thrombosis.
**Patient 5**
A 70-year-old woman referred for the evaluation of leukocytosis and thrombocytosis without splenomegaly. The hematocrit was 44%, the leukocyte count was 13,500/mL, and the platelet count was 1,300,000/mL. The red cell mass was elevated at 35.8 mL/kg, with an expected range of 20–30 mL/kg. The plasma volume was 45.4 mL/kg with an expected value of 40 mL/kg, establishing polycythemia vera as the cause of the abnormal blood counts.

**Table 2:** Critique of the WHO diagnostic criteria for polycythemia vera.

Major Criteria	Critique
1. Hemoglobin > 16.5 g/dL in men Hemoglobin > 16.0 g/dL in women	Hemoglobin is a red cell product; its level varies with body iron stores and it does not reflect the red cell mass
or,	
Hematocrit > 49% in men Hematocrit > 48% in women	There is no safe hematocrit when polycythemia vera is a diagnostic consideration
	The red cell count and the MCV have been omitted even though microcytic erythrocytosis provides a clue to absolute erythrocytosis
or,	
Increased red cell mass (RCM) *	
2. BM biopsy showing hypercellularity for age with trilineage growth (panmyelosis), prominent erythroid, granulocytic, and megakaryocytic proliferation with pleomorphic, mature megakaryocytes (differences in size)	MPN bone marrow histology is not diagnostic for PV
3. Presence of JAK2 V617F or JAK2 exon 12	A quantitative allele burden is mandatory as normal individuals can have a positive qualitative assay
Minor criterion	
Subnormal serum erythropoietin level	A low erythropoietin assay is not diagnostic for PV
Diagnosis of PV requires meeting either all 3 major criteria, or the first 2 major criteria and the minor criterion ^†^	Curiously, PV is a panmyelopathy but leukocytosis, thrombocytosis and splenomegaly have been omitted as diagnostic criteria

* More than 25% above mean normal predicted value. ^†^ Criterion number 2 (BM biopsy) may not be required in cases with sustained absolute erythrocytosis: hemoglobin levels 18.5 g/dL in men (hematocrit, 55.5%) or 16.5 g/dL in women (hematocrit, 49.5%) if major criterion 3 and the minor criterion are present. However, initial myelofibrosis (present in up to 20% of patients) can only be detected by performing a BM biopsy; this finding may predict a more rapid progression to overt myelofibrosis (post-PV MF). There are no data substantiating this claim.

## Glossary

ARCH	Age-related clonal hematopoiesis is an accelerated version of CHIP, occurring after the age of 60 years.
ACVR1	Activin A Receptor Type 1, also known as ALK-2, is a member of the BMP/TGF-β receptor family.
CHIP	Clonal hematopoiesis with an indeterminate potential is a syndrome characterized by the acquisition of gene mutations by hematopoietic stem cells (HSCs) as they age, with a MAB of ≥2%. Most of these mutations are not potentially deleterious, but a few are, including *DNM3T*a, *ASXL*1, *TP*53, *TET*2, and *JAK*2 V617F.
Contiguous gene syndrome	Contiguous gene syndrome (CGS) is a de novo or treatment-related genetic disorder in which chromosomal copy number changes involving multiple neighboring genes occur, causing del(5q) or –7/del(7q) or complex karyotypes with an adverse prognosis.
Erythremia	An archaic term for polycythemia vera.
Genotype	The genetic basis for phenotype.
Homologous recombination	A mechanism for exchanging genes between similar DNA molecules.
Latency	The time between a biologic change, such as the acquisition of a gene mutation, and the phenotypic expression of that mutation.
LOH	Loss of heterozygosity occurs when a single allele or a larger segment of DNA is lost from a normal chromosome by one of several mechanisms, including gene mutation, deletion, or mitotic recombination, and most commonly in MPNs by uniparental disomy (UPD).
MAB	Mutation allele burden, also known as the VAF, or variant allele fraction, is the percentage of chromosomes in a cell that carries a particular gene mutation. Either one allele (heterozygosity for the mutation) or both alleles of the gene (homozygosity for the mutation) can be involved.
Newtonian fluid	A fluid whose shear stress from flow is linearly correlated with its rate of change in deformation over time.
Next-generation sequencing	Massive, parallel DNA sequencing, also known as NGS, allows the interrogation of the human genome accurately and rapidly to detect gene mutations.
Nitric oxide scavenging	When nitric oxide in the blood stream is bound by either free hemoglobin or red cells, vasoconstriction and increased platelet adherence to the vascular endothelium occur.
Non-Newtonian fluid	The behavior of a fluid that deviates from Newton’s law of viscosity. As an example, blood is more viscous when flowing slowly than when it is flowing rapidly.
Pagophagia	A form of Pica in which disordered gustatory behavior focuses on eating ice.
Phenotype	The observable features of an individual.
Penetration	The extent to which a genetic change is expressed.
Phenotypic mimicry	When appearance is clinically deceiving, which is a particular problem with MPNs, where the same mutation causes two or three different disease phenotypes.
Reactive oxygen species	Reactive oxygen species, such as superoxide, singlet oxygen, hydroperoxide, or hydroxyl radicals, are unstable and can damage DNA, RNA, or proteins.
Ribonucleotide reductase	This enzyme catalyzes the reduction in ribonucleotides to deoxyribonucleotides for use in DNA synthesis. It is a target for hydroxyurea, which destroys its tyrosyl free radical.
JAK2 pseudokinase domain	This is a region of the JAK2 protein that lacks the necessary residues to be a kinase, but can act as an inhibitor of the protein’s tyrosine kinase domain.
Uniparental disomy	Acquired uniparental disomy (UPD), a common feature of PV, is a mitotic recombination resulting in an exchange of genes inherited from one parent on the same segment of one of two homologous chromosomes, causing the copy neutral loss of heterozygosity that is undetectable by cytogenetics.
